# The Impact of the Unstructured Contacts Component in Influenza Pandemic Modeling

**DOI:** 10.1371/journal.pone.0001519

**Published:** 2008-01-30

**Authors:** Marco Ajelli, Stefano Merler

**Affiliations:** 1 Fondazione Bruno Kessler, Trento, Italy; 2 Information Engineering and Computer Science Department, University of Trento, Italy; Yale University, United States of America

## Abstract

**Background:**

Individual based models have become a valuable tool for modeling the spatiotemporal dynamics of epidemics, e.g. influenza pandemic, and for evaluating the effectiveness of intervention strategies. While specific contacts among individuals into diverse environments (family, school/workplace) can be modeled in a standard way by employing available socio-demographic data, all the other (unstructured) contacts can be dealt with by adopting very different approaches. This can be achieved for instance by employing distance-based models or by choosing unstructured contacts in the local communities or by employing commuting data.

**Methods/Results:**

Here we show how diverse choices can lead to different model outputs and thus to a different evaluation of the effectiveness of the containment/mitigation strategies. Sensitivity analysis has been conducted for different values of the first generation index *G_0 _,* which is the average number of secondary infections generated by the first infectious individual in a completely susceptible population and by varying the seeding municipality. Among the different considered models, attack rate ranges from 19.1% to 25.7% for G*_0_* = 1.1, from 47.8% to 50.7% for *G_0_* = 1.4 and from 62.4% to 67.8% for *G_0_* = 1.7. Differences of about 15 to 20 days in the peak day have been observed. As regards spatial diffusion, a difference of about 100 days to cover 200 km for different values of *G_0_* has been observed.

**Conclusion:**

To reduce uncertainty in the models it is thus important to employ data, which start being available, on contacts on neglected but important activities (leisure time, sport mall, restaurants, etc.) and time-use data for improving the characterization of the unstructured contacts. Moreover, all the possible effects of different assumptions should be considered for taking public health decisions: not only sensitivity analysis to various model parameters should be performed, but intervention options should be based on the analysis and comparison of different modeling choices.

## Introduction

Facing the potentially devastating impact of a new influenza pandemic is a major priority of national and international public health agencies. As a consequence, the need clearly arises to develop suitable measures of containment/mitigation.

While the spatiotemporal epidemic dynamics and the assessment of some containment or mitigation strategies, as mass vaccination [1] or border restriction [2], can be predicted by employing classical compartmental models (SIR o SEIR models, possibly with age and/or geographic component), the evaluation of realistic, individually targeted, public health intervention strategies, as antiviral prophylaxis of household or school/workplace contacts of index cases, in turn requires highly detailed models. Spatially explicit models provide a plausible system in which the precise spatial location of individuals and movement patterns can be employed to evaluate intervention options [3].

Spatial models can be broadly divided into three major categories: patch models, distance-based models, network models. In patch models, the force of infection (FOI) received by an individual living in a patch (e.g., a town) depends on the distance between the patch of residence and the patches of the infectious individuals. As a consequence, patch models are not explicitly Individual Based Models (IBM) and all the members of a patch receive the same FOI. Distance-based models are explicitly IBM where individuals are assigned a precise location and the FOI is a decreasing function of the distance between susceptible and infectious individuals. To reduce the computational burden, it can be set to 0 for distances greater than a given threshold. In network models, which are explicitly IBM, individuals are connected to other individuals by co-locating them into groups (e.g., households, schools and workplaces, etc.). Additionally, the members of a group can be not equally well connected. For instance, in large schools or workplaces subgroups of individuals, representing classmates or close colleagues, can be more strongly connected. The FOI received by a susceptible individual is non zero when he/she shares an arc with a infectious individual.

IBM are currently considered the best tools since they allow the explicit representation of the actual locations where intervention measures will be implemented to reduce transmission. In [4] and [5] a network model (explicitly modeling households, schools and workplaces) is coupled to a distance based model (describing the random component of the transmission) for assessing the effectiveness of containment strategies in South-East Asia [4] and of mitigation strategies in US and UK [5]. In [6] a network model is employed for assessing the effectiveness of containment strategies based on antiviral prophylaxis in a typical American community. In [7] a network model where individuals have occasional contacts (of fixed average size 100) with individuals living in the commuting patch (for work) is employed for assessing the effectiveness of containment strategies in South-East Asia. In [8], a network model where individuals take occasional long distance trips is employed for assessing the effectiveness of mitigation strategies in US.

As regards control, last generation models allow the evaluation of intervention measures targeted at individual level, in locations such as households, schools, workplaces, transportations and hospitals which are among the most important routes of influenza transmission. As regards transmission, these models allow the most detailed representation of social contacts between individuals. They are also suitable for modeling smallpox outbreaks and to evaluate the impact of containment strategies [9], [10].

IBM represent the more obvious way to relax assumptions that were considered mandatory in traditional mathematical models of diseases, such as the assumption of homogeneous mixing, which most of mathematical models are based on. As a first step in the construction of an IBM, contacts are progressively “structured'” by co-locating individuals into the diverse environments where they are expected to have contacts, namely households, school, workplaces, commuting and public transportations and so on. However, one readily realizes that most epidemics initiating from a single focus would die out or would not travel, unless a degree of (pseudo)-randomness in contacts, perhaps small, is allowed. Though largely unknown, the impact of the unstructured component on the epidemics dynamics is not necessarily small: for instance, many current flu models are based on the explicit assumption that unstructured contacts account for about 1/3 of the total risk of infection per unit time [4],[5]. Moreover, unstructured contacts prevent epidemics to die out and allow more structured contacts to amplify the epidemics themselves, thus leading to potentially devastating outbreaks. The currently available generation of infectious diseases IBM have achieved a sophisticated descriptions of the structured component of contacts, but the unstructured component continues to be loose because of scarce information on unstructured ones which are however the sustaining factor. What concretely happens is that unstructured contacts are modeled in a residual way, mainly reflecting the researcher feeling, and at best are left as free simulation parameters.

The purpose of this work is to evaluate to what extent different modeling strategies for unstructured contacts can affect pandemics prediction and control. Motivated by the issue of modeling interventions aimed at containing a national flu pandemic, we provide a comparison of various alternatives to model the unstructured component. These alternatives include the main approaches proposed in the literature and comprehend some new techniques. In particular, we keep the structured component fixed, and we vary the unstructured one, looking at the implications in terms of the major epidemic outputs, as fade-out and extinction probabilities, spatial traveling, attack rates, and proportion of infected individuals by age.

The modeling framework adopted for the comparison, particularly for the structured component, is represented by the recently developed IBM used for pandemic prediction and control in Italy. Three main approaches are considered to model the unstructured component: a spatially explicit model depending on a parametric kernel function of the distance, with asymptotic power-law form [4], [5]; a model where random contacts are chosen in the local communities [8]; a model where random contacts are defined on the basis of commuting data, as suggested in [3]. For ease we term the three models as models **S**, **L** and **M** respectively. Moreover, we also included occasional long-distance trips **T** (as in [8]) in models **L** and **M**, called now **L+T** and **M+T** respectively.

## Results

More than 1,000,000 experiments were run to evaluate how the different approaches to modeling unstructured contacts can affect the spatiotemporal epidemic dynamics. For each considered model, different model instances were realized by varying the *first generation index G_0_* defined as the average number of secondary infections generated by the first infectious individual during the entire infectious period in a completely susceptible population (more details are given in Methods): *G_0_* values of 1.1, 1.4 and 1.7 were considered to simulate low to high transmission scenarios. We compared a variety of summary measures such as the probability of having a large outbreak, the epidemic evolution (attack rate, basic reproductive number, peak day, proportion of infected by age) and the spatial diffusion, i.e., the average distance from the seed area for individuals infected since the start of the epidemic as a function of time. For comparison's sake, all the simulations were seeded with only one infected individual, even though a pandemic influenza in a European country will be very likely sustained by mechanisms of case importation, e.g., by international travels [2], [11], [12], [13]. Different seeding municipalities were chosen to take into account the role played by the demographic size, density and geographic location of the seeding zone; we considered large cities, small/medium size towns, isolated villages, and, as an extreme case of isolated seeding region, islands.

The final attack rate of the considered models is significantly different (see Table 1) and it ranges from 19.1% to 25.7% for *G_0_* = 1.1, from 47.8% to 50.7% for *G_0_* = 1.4 and from 62.4% to 67.8% for *G_0_* = 1.7. No substantial differences are observed by varying the seeding municipality. The introduction of occasional long-distance trips substantially decreases the final attack rate of both the **M** and the **L** models. In fact, in our implementation, transmission is not allowed within household and within school/workplace during long-distance trips.

**Table 1:** Final attack rates

Model/*G_0_*	1.1	1.4	1.7
**M**	25.7 (0.029)	50.7 (0.014)	64.6 (0.011)
**M+T**	21.4 (0.040)	47.7 (0.016)	62.4 (0.011)
**L**	26.9 (0.031)	50.7 (0.016)	67.6 (0.011)
**L+T**	22.9 (0.035)	47.8 (0.018)	65.7 (0.011)
**S**	19.6 (0.077)	48.6 (0.039)	64.8 (0.017)

Final attack rates (with standard deviation) of the different models considered for different *G_0_* values.

The basic reproductive number *R_0_* of the simulated epidemics is calculated as in [8], [14] (see Methods). The observed *R_0_* values, among all the considered models, do not vary more than 0.07, 0.11 and 0.08 for *G_0_* = 1.1, 1.4 and 1.7 respectively (see Table 2). Note that *R_0_* is systematically larger than the average number of secondary cases generated by the primary infection in a wholly susceptible population, as observed in [8].

**Table 2:** Basic reproductive numbers

Model/*G_0_*	1.1	1.4	1.7
**M**	1.34 (0.018)	1.78 (0.010)	2.18 (0.011)
**M+T**	1.29 (0.022)	1.71 (0.010)	2.11 (0.013)
**L**	1.34 (0.022)	1.73 (0.011)	2.16 (0.016)
**L+T**	1.29 (0.025)	1.67 (0.011)	2.10 (0.011)
**S**	1.27 (0.029)	1.72 (0.013)	2.14 (0.008)

Basic reproductive numbers *R_0_* (with standard deviation) of the different models considered for different *G_0_* values.

**Figure 1:**
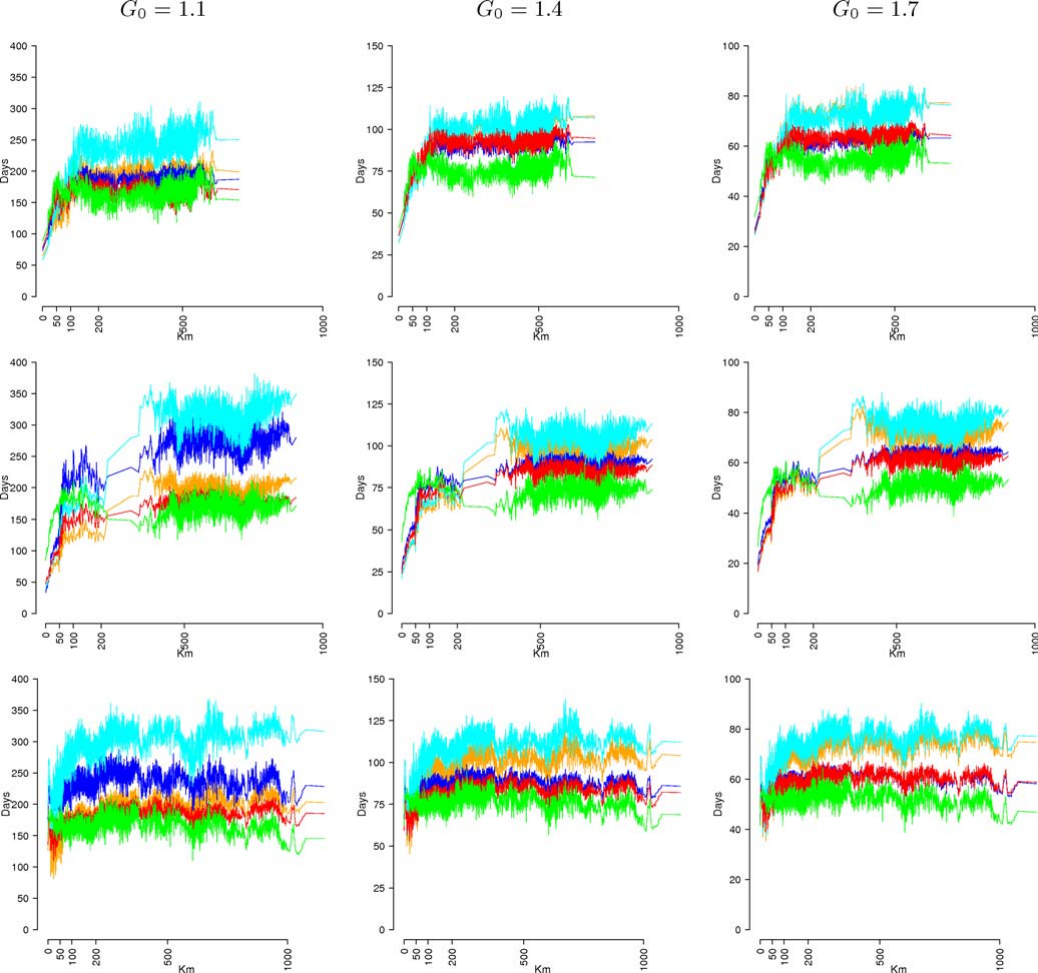
Spreading time from the seeding municipality as a function of the distance for different values of *G_0_* and different seeding municipalities: Rome (first row), Cagliari (second row), a small isolated village (third row). Model M in orange, M+T in red, L in cyan, L+T in blue, and S in green.

**Figure 2:**
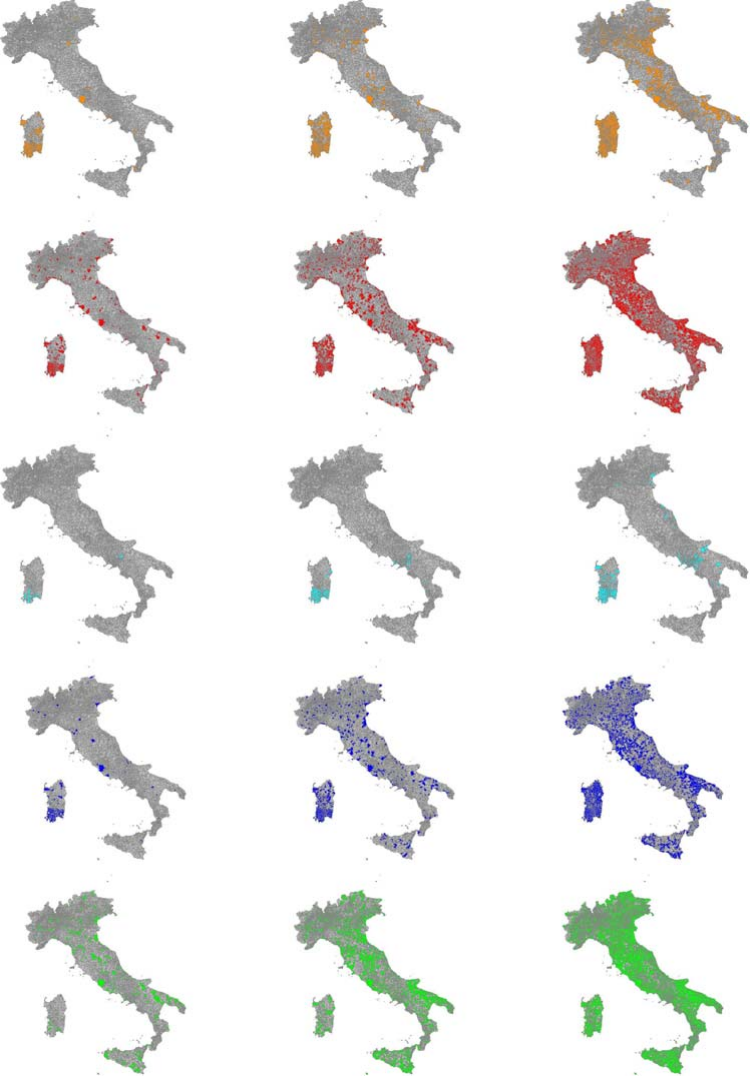
Spatiotemporal dynamics at 40 (left), 50 (center) and 60 (right) days. Infection is seeded in Cagliari (Sardinia island) and *G_0_* = 1.7. Coloured areas (model M in orange, M+T in red, L in cyan, L+T in blue, and S in green) indicate presence of at least one infected, infectious or removed individual.

Significant differences can be detected in the spatial diffusion of the epidemic (see Figure 1). For *G_0_* = 1.1, **L** models are spread systematically more slowly than the respective **M** models (with difference of about 100 days to cover 200 km). In fact, the set of unstructured contacts as considered in **M** models includes individuals living in or traveling to the same municipality where the individuals travel to, thus inducing a higher probability of exporting the epidemic. Due to the specific choice of kernel function and parameters, **S** models are spread systematically more quickly (with difference of about 100 days to cover 200 km with respect to **M** models). However, alternative choices of kernel function and parameters can lead to different model outputs. The behavior of **L** and **M** models tends to be similar when increasing the first generation index, while the **S** models are systematically the fastest. Not surprisingly, models including long-distance trips **M+T** and **L+T** spread quite faster than the respective **M** and **L** models (even though their attack rate is systematically lower), independently from the first generation index and the seeding municipality. Furthermore, the observed pattern of spatial spread strongly depends on the seeding region. For instance, when the epidemic is seeded in a small, isolated village, no clear pattern of diffusion is observable (especially for **S** models) since the epidemic is more likely to spread towards far, large cities than towards close, small size municipalities (see Figure 1, third row). At a given distance, the variability observed in the time of epidemic arrival is basically determined by the variability in the population size of the arrival municipalities. Trivially, on average, the epidemic is very likely to spread first towards large population municipalities than towards small, isolated municipalities. When infection is seeded in very isolated regions, as Sardinia island, the models behave quite differently (see Figure 1, second row). Basically, in **M** and **L** models the epidemic is spread on the entire island before being spread out to the rest of Italy. A similar behavior is observed in **M+T** and **L+T** models, even though it is not so pronounced, while in **S** model the epidemic is spread out in the first phase (see also Figure 2 and Movie S1). In fact, only a very small fraction of workers and students commutes to or from the island, greatly reducing the set of contacts outside the island in **M** and **L** models, while this is not the case for **S** models. However, note that the kernel parameters of the spatially explicit model were chosen on the basis of commuting data (see Text S1). While this is a reasonable choice for assigning commuting destination, it is unclear whether this is the best choice for modeling the spatial spread of an epidemic through unstructured contacts. Completely different behaviors are to be expected when adopting different kernel shapes. Although the spatially explicit model is flexible, it requires detailed data, both demographic and epidemiological, for choosing the optimal kernel and kernel parameters.

**Figure 3:**
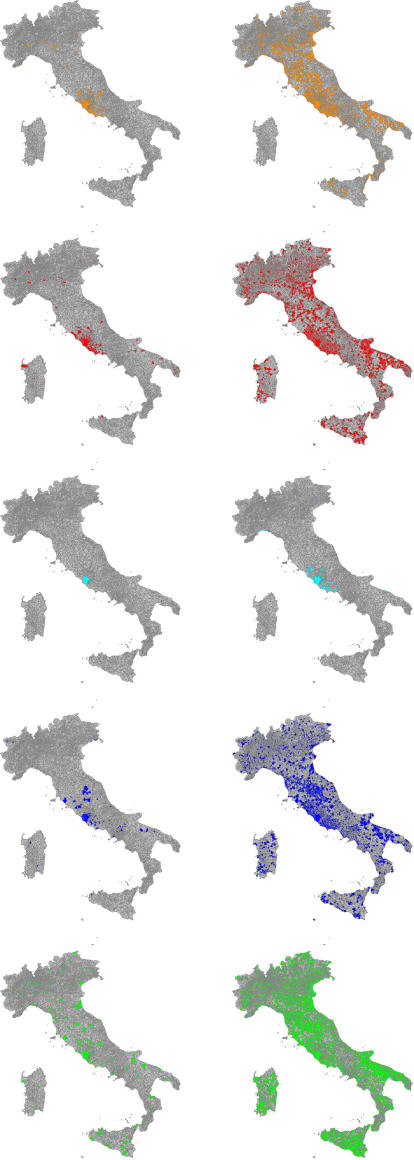
Spatiotemporal dynamics at 40 (on the left) and 60 (on the right) days, roughly corresponding to the begin and the end of the exponential growth phase. Infection is seeded in Rome and *G_0_* = 1.7. Colored areas (model M in orange, M+T in red, L in cyan, L+T in blue, and S in green) indicate presence of at least one infected, infectious or removed individual.

**Figure 4:**
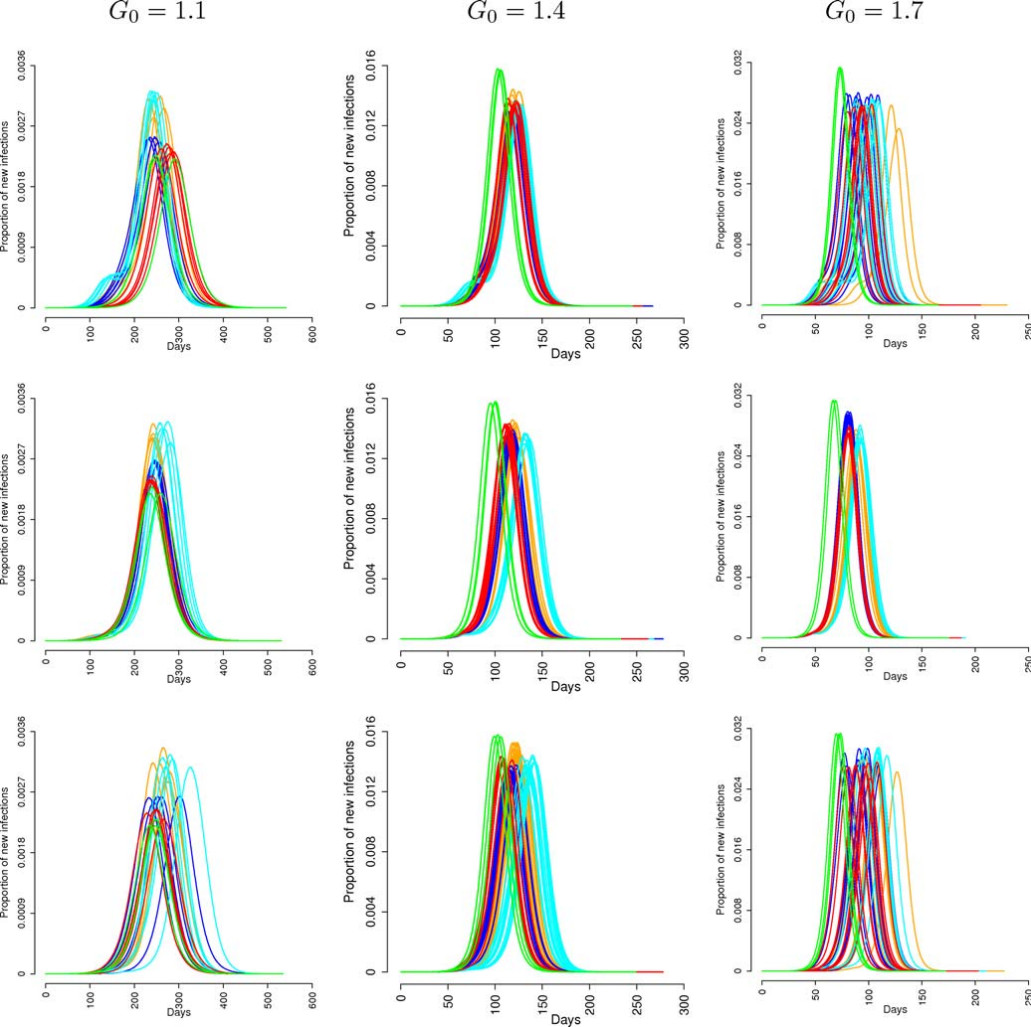
Number of daily cases for different values of the first generation index and different seeding municipalities: Rome (first row), Cagliari (second row), a small isolated village in the north of Italy (third row). Simulation are initialized with 30 infected individuals, to reduce the stochastic variability observed in first days of the epidemic. Models are: M in orange, M+T in red, L in cyan, L+T in blue, and S in green.

Significant differences are observed in the peak day (see Table 3). In particular, for large values of *G_0_* (*G_0_*≥1.4) the epidemic peak of **S** models occurs systematically earlier than **M** and **L** models (with differences of about 15 to 20 days for different values of the first generation index). Since in **S** models the epidemic is spread much more quickly, new infection foci occur simultaneously in many different regions, thus inducing a spatial synchronization of the epidemic (see Figure 3 and Movie S2). No substantial differences are observed by varying the seeding region (see Table 4). For *G_0_* = 1.1, no significant differences are observed between **M** and **S** models, while, on average, the peak day of **L** models occurs later than **M** and **S** models. This is due to the several simulations behaving very differently from all the others (and independently from the seeding region), characterized by a very long initial phase and giving rise to a high standard deviation. In fact, for low values of the first generation index, **L** models are less likely to spread out the epidemic because of the reduced set of unstructured contacts. Not surprisingly, the introduction of occasional long-distance trips significantly anticipates the epidemic peak in both the **M** and **L** models (5 to 10 days earlier than the respective **M** and **L** models). See also Figure 4 where the number of cases in time of the different models are reported for different seeding municipalities and different first generation indices.

**Table 3:** Peak day

Model/*G_0_*	1.1	1.4	1.7
**M**	287.1 (22.0)	143.7 (11.3)	104.5 (6.7)
**M+T**	294.9 (29.0)	137.9 (10.2)	98.9 (7.2)
**L**	439.3 (127.4)	153.6 (11.5)	107.3 (7.1)
**L+T**	407.6 (163.7)	142.9 (10.7)	99.3 (7.7)
**S**	302.0 (33.8)	127.4 (10.6)	90.3 (7.7)

Peak day (with standard deviation) of the different models considered for different *G_0_* values.

**Table 4:** Peak day for different seeding municipality

*G_0_*	Municipality	M	M+T	L	L+T	S
**1.1**	**Rome**	287.2 (20.6)	289.3 (25.8)	429.5 (129.7)	396.3 (153.6)	299.0 (33.6)
	**Cagliari**	288.4 (27.3)	286.2 (26.2)	448.2 (126.5)	448.2 (174.1)	300.9 (33.7)
	**Luserna**	286.4 (13.8)	294.6 (26.4)	421.5 (143.7)	380.8 (144.9)	298.3 (38.0)
	**Turin**	286.2 (20.5)	288.9 (25.4)	427.9 (130.3)	388.3 (148.2)	298.8 (31.3)
	**Vieste**	287.1 (22.1)	294.9 (29.0)	439.4 (127.5)	407.7 (163.8)	302.1 (33.8)
**1.4**	**Rome**	144.9 (12.4)	136.7 (10.2)	154.4 (10.5)	142.1 (10.7)	127.9 (11.8)
	**Cagliari**	142.5 (9.6)	136.0 (10.2)	153.5 (9.4)	142.0 (11.0)	126.1 (12.3)
	**Luserna**	145.9 (12.7)	134.2 (8.4)	158.0 (10.3)	141.1 (9.9)	129.2 (11.5)
	**Turin**	143.6 (11.9)	136.6 (10.2)	155.0 (12.0)	142.8 (11.5)	127.6 (11.3)
	**Vieste**	143.8 (11.4)	138.0 (10.3)	153.6 (11.6)	143.0 (10.8)	127.4 (10.6)
**1.7**	**Rome**	106.1 (6.6)	97.8 (6.2)	108.7 (6.5)	98.5 (7.5)	89.7 (6.4)
	**Cagliari**	105.5 (5.6)	98.0 (6.2)	109.7 (6.3)	99.7 (7.5)	87.9 (6.1)
	**Luserna**	106.0 (6.3)	97.3 (5.9)	109.5 (6.0)	98.3 (7.3)	88.6 (6.8)
	**Turin**	104.9 (6.9)	98.0 (6.8)	107.9 (7.3)	98.9 (7.7)	89.6 (6.2)
	**Vieste**	104.5 (6.7)	99.0 (7.3)	107.4 (7.1)	99.4 (7.8)	90.4 (7.7)

Peak day (with standard deviation) for different seeding municipality and different values of the first generation index *G_0_*. Roma is the largest city of Italy (2,546,804 inhabitants), located in the central Italy; Cagliari is a city (164,249 inhabitants) in the Sardinia island; Luserna is a small isolated village (297 inhabitants) in the northern of Italy; Turin is a big city (865,263 inhabitants), located in the northern Italy; Vieste is a small town (13,430 inhabitants) in the southern Italy.

**Figure 5:**
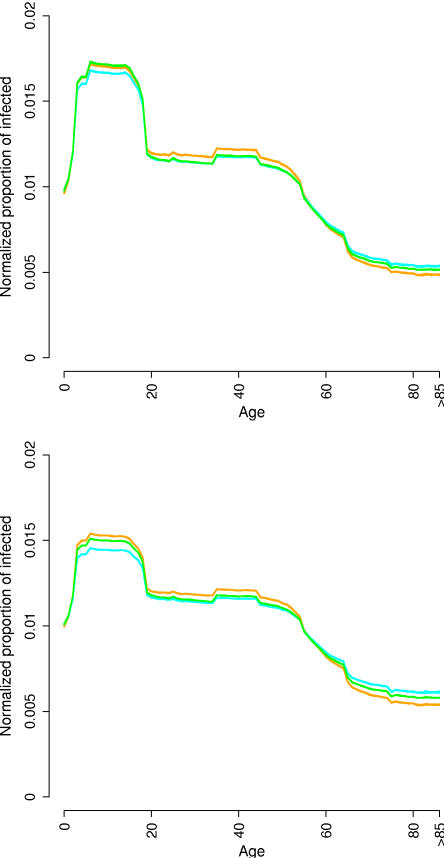
Proportion of the infected population by age for *G_0_* = 1.4 (top) and *G_0_* = 1.7 (bottom). Models M in orange, L in cyan and S in green.

Differences are also observed in the proportion of infected by age (see Figure 5). In order to compare the different models, the curves are normalized, and we consider the indicator 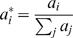, where *a_i_* is the proportion of infected of age *i*. Independently from the first generation index, the proportion of infected generated by **M** models in individuals older than 65 years is lower than for other models, while the opposite behavior is observed for individuals younger than 65 years. In terms of unnormalized proportion of infected, differences of 5% to 10% are observed in the older individuals for *G_0_* = 1.7. In fact, in **M** models the set of unstructured contacts of infectious individuals proportionally includes a larger number of traveling individuals (i.e., with age between 3 and 65 years). Consequently, this latter class of individuals is proportionally more exposed to contacts with infectious ones. This is not the case for age independent unstructured contacts models, as **S** and **L** models. In [4], [5], [8], the authors introduce additional parameters to make the unstructured contacts dependent on age, while for **M** models this is obtained in a natural way. The slightly larger proportion of infected observed in individuals aged 35–45 is due to the structured component of the contacts: in fact, they have a higher probability of living with individuals aged 3–18, the most infected class.

Finally, no substantial differences are detected in the probability of observing a large outbreak for *G_0_* = 1.1 and *G_0_* = 1.4 (see Table 5). For *G_0_* = 1.7, the probability of a large outbreak in **S** models is larger than that observed for the other models.

**Table 5:** Probability of a large outbreak

Model/*G_0_*	1.1	1.4	1.7
**M**	0.184 (0.050	0.449 (0.027)	0.572 (0.060)
**M+T**	0.184 (0.096)	0.446 (0.072)	0.598 (0.055)
**L**	0.192 (0.047)	0.460 (0.079	0.628 (0.023)
**L+T**	0.154 (0.040)	0.464 (0.086)	0.661 (0.013)
**S**	0.201 (0.047)	0.474 (0.007)	0.662 (0.021)

Probability (with standard deviation) of a large outbreak for the different models considered and for different *G_0_* values.

In Text S1, sensitivity analysis on the effect of varying the number of travel days in models **M+T** and **L+T** and on the effect of varying the spatial kernel in models **S** is carried on (see Table S1, Table S2 and Table S3).

## Discussion

In principle, it would be possible to improve the characterization of structured contacts, for instance by employing data on contacts on neglected but important activities, such as leisure time, sport mall, restaurants, etc. and time-use data to provide useful information for parameterizing IBM. Such information starts being available (see the EU project Polymod) and it will be mandatory to integrate it in the next generation of IBM. However, it is not possible to take into account all the possible sources of infections. In fact, this would mean tracing all the possible contacts (which in turn requires to model all the places where these contacts occur, how much time is spent in each place, etc.), to establish the “type” of contacts (e.g., skin to skin or indirect) of each individual, which is unfeasible. It is thus required to consider in the models a source of infection accounting for pseudorandom contacts.

The scenarios emerging from the conducted experiments in terms of final attack rate, spatial spread, epidemic peak day and proportion of infected by age are quite heterogeneous. In particular, epidemics generated by the spatially explicit model spread much more quickly than those simulated by all the other models, regardless of first generation index and seeding region. Also, the epidemic peak occurs systematically earlier, probably because of spatial synchronization effects. Defining unstructured contacts on the basis of commuting data rather than randomly choosing them in the local communities results in a faster epidemic, especially for lower values of the first generation index, in terms of both spatial diffusion and peak day. The effects of occasional long-distance trips are the speeding up of the spatial diffusion and the decreasing of the cumulative attack rate. The proportion of infected by age is also significantly different. Specifically, the proportion of infected in the younger and adult age groups is larger in the models where random contacts are defined on the basis of commuting data while the proportion of infected in the older age groups is lower. No significant differences are observed in the probability of having a large outbreak, especially for small first generation indices.

Wide differences in the models' outputs can result in different evaluations of the effectiveness of the containment/mitigation strategies and they would seriously undermine the usefulness of our models, thus urgently calling for field work aimed at filling this data-gap. In fact, even though the containment strategies are in general based on the structured part of the contacts (social distancing measures, e.g. school and workplaces closure, antiviral prophylaxis on a contact tracing basis), the way we choose to model the unstructured part of the contacts can lead to very different scenarios.

A detailed analysis of the implications in terms of containment strategies evaluation is beyond the aim of this work. A few considerations can be drawn, anyway. Trivially, the cumulative attack rates are quite different, even though the models are initialized in the same way, thus leading to different evaluations of the effectiveness of the same containment measure. More specifically, the difference observed in the peak day can result in different evaluations on the effectiveness of vaccination campaigns. For instance, in a mass vaccination campaign against a pandemic with *G_0_* = 1.4, by fixing target population at 60%, vaccine efficacy at 70% and vaccine availability at 4 months after the first national case, the number of avoided cases is, on average, 24.4 millions for the **M** model, 20.2 millions for the **M+T** model, 26.8 millions for the **L** model, 22.7 millions for the **L** model and 13.4 millions for the **S** model. Moreover, ignoring the variations in the proportion of infected by age can result in wrong decisions when optimizing the choice of the target population for a vaccination campaign. Furthermore, the variability of the spatial spread can influence the evaluation of strategies based on geographical targeting. We can mention the choice of the dimension of quarantine areas, the effectiveness of antiviral prophylaxis on a geographical basis and the timing for closing schools and workplaces: for instance, close them all simultaneously or wait for a few cases to arise? While the observed differences could not drastically undermine the results in terms of feasibility of the considered interventions (the principal objective of many independent studies), nevertheless they could be relevant in terms of optimality.

Wide differences in the models' outputs can result in different evaluations of the effectiveness of the containment/mitigation strategies. Consequently, all the possible effects of different assumptions should be considered for taking public health decisions: not only sensitivity analysis to various model parameters should be performed, but intervention options should be based on the analysis and comparison of different modeling choices, as it happens in different fields, e.g. global climate change, where uncertainty in the models themselves and in input parameters is a critical factor.

We conclude remarking that unlike what shown in most of the literature [3], [4], [5], [8], no supercomputing techniques have to be employed to perform this kind of simulations on a national scale (57,000,000 of individuals), making them feasible for a standard workstation; our implementation of the five model takes less than 3Gb RAM and a single simulation takes just a few minutes.

## Methods

IBM allow a highly detailed treatment of the majorly problematic issues in modeling the transmission dynamics and the control of human-to-human infectious diseases. They are specifically suitable for modeling influenza pandemic since they allow the modeling of the contacts of the specific places where groups of individuals spend much of their time together, i.e. households, schools and workplaces. Namely, it is crucial to develop models accounting for these transmission sources and allowing the evaluation of specific, place-related intervention strategies. Of course, while this modeling effort is required for analyzing human-to-human airborne transmitted diseases, it is not required when dealing with diseases transmitted by other routes, as sexually transmitted diseases (e.g., AIDS) or orofecal transmitted infections (e.g., Hepatitis A). To date, national scale, spatially explicit IBM do not account for dynamic contact networks, where individuals are born, grow up, mate, produce offspring, and die. Thus, they are currently less suitable for modeling endemic diseases, as measles or seasonal flu. Moreover, they do not account for spontaneous behavioral changes in response to a pandemic and do not take into account the social acceptance of eventual restriction measures, as quarantine, school and workplace closure and travel restrictions. Specific work should be devoted to bridge these gaps.

The models considered in this work are stochastic, individual based, discrete-time, SEIR simulations. Infection can be spread by three main contact routes: within households, within schools and workplaces, which are the channels we call structured, and by random contacts in the population, termed unstructured. The main ingredients are (A) a socio-demographic model (kept fixed in all the considered models), in which individuals are co-located in households, schools and workplaces on the basis of census and commuting data and (B) a transmission model describing the temporal evolution of the flu epidemic in the considered study area (Italy). Transmission within households, schools and workplaces is the same throughout all the considered models, while we adopt different approaches for the transmission by unstructured contacts.

Individual based models can be analyzed by employing analytical tools (e.g., by eliminating spatial heterogeneity or by eliminating individual variability) [15] or by performing intensive simulation studies to evaluate sensitivity to various model parameters, as in [4]–[8]. The latter is the approach followed in this work.

### Structured contacts

Population data of Italy (56,995,744 individuals) are obtained from census data (382,534 census sections) as of 2001 and they are hierarchically grouped by municipalities (8,101), provinces (103) and regions (20), according to the administrative borders of the study area. This choice is determined by the availability of nationwide commuting data organized at the same level of detail (see Figure S1).

Census data on age structure and household type and size are jointly used with survey data to randomly assign age and to co-locate individuals in households. Nine different types of households are considered, e.g. single with or without children and couple with or without children. For each household type, data on size and age of the household head are taken into account to generate households (see Table S4, Table S5, Figure S2, Figure S3b and Figure S3c).

Demographic, school and industry census data are used for randomly assigning an employment category to each individual on the basis of age. The Italian population is stratified as follows: 20,559,595 workers (for transmission's purposes, the 862,552 teachers are included in the school channel), 11,360,556 students and 25,084,274 unemployed or retired. Students are deterministically assigned to a specific school type (6 types, from nursery school to university) on an age basis. Workers are assigned to a random workplace type (7 types, depending on the number of employees) or to a school (see Figure S3d).

Commuting destination are assigned so to fit available commuting data. In particular, for each municipality the proportions are available of individuals older than 15 years old working or attending school a) in the municipality of residence or traveling b) within the province they live in, c) outside the province but within the region, d) outside the region. We allow younger students to travel only within the province they live in. More details on the structured component of the contacts are given in Text S1 (see also Figure S3e and Figure S3f).

### Unstructured contacts

Here we define unstructured any contact which is not a household or workplace contact and we consider the following five different models of transmission by unstructured contacts (details on these models are given in Text S1).

Model **S**: unstructured contacts through the whole space by a distance-based model. Each individual is in contacts with every other individual in the population, with probability (decreasing with the distance) given by a specific kernel function (see Figure S4).

Model **L**: unstructured contacts within the municipality the individual lives in.

Model **M**: unstructured contacts within the “commuting community'” the individual belongs to. In particular, for individuals who study or work in the same municipality they live in, the social network consists of other inhabitants of the same municipality and those who commute to this municipality. For individuals traveling outside the municipality of residence, the social network consists of the inhabitants and commuters of both depart and arrival municipality (see Text S1).

Moreover, we consider two additional models including occasional long-distance trips [8] in models **L** and **M**, called **L+T** and **M+T** respectively. In particular, all individuals are assumed to spend in average 10 days (randomly chosen) per year in a community other than that of residence and school/work. In these periods, within household, school and workplace transmission is not allowed.

### Transmission model

Any susceptible individual *i*, at any time of the simulation has a probability 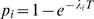 of becoming infected, where *T* = 0.5 days is the time-step of the simulation and *λ_i_* is the instantaneous risk of infection. The latter is the sum of the risks coming from the three source of infections: contacts with infectious members of the household, contacts with infectious individuals working in the same workplace or attending the same school, random contacts with infectious individuals in the population. Details on the Transmission model are given in Text S1.

### Transmission rates

Comparison (in terms of spatiotemporal dynamics, attack rate, proportion of infected per age other relevant features) among models is meaningful only for simulations sharing the same first generation index *G_0_*. We recall that this is the average number of secondary infections generated by the first infected individual, during his entire infectious period, in a completely susceptible population.

In traditional models the simplest choice would be to fix the basic reproduction number *R_0_* (see [1], [16]), which can be estimated by approximating the slope of the cumulative number of cases during the exponential growth phase of the epidemic. The difference between first generation index and basic reproduction number lies on the fact that the former is determined only by the first generation of infection while the latter emerges after the underlying next generation operator is applied for a sufficiently large number of generations. Our choice is motivated by the simplicity of the *G_0_* computation, in opposition to the difficulty in appropriately calculating *R_0_* for individual based models (see [8], [17]). Moreover, by adopting the first generation index as comparison indicator, all the models are initialized in the same way.

Three different scenarios are investigated, corresponding to *G_0_* = 1.1;1.4;1.7. All the simulations are initialized with only one infected individual, yielding a completely susceptible population. Estimate of the transmission rates in the different transmission places (household, school/workplace and community) leading to the chosen *G_0_* value is done by keeping trace of number and place of the secondary infections. A reference model (the **M** model for instance) is chosen and transmission coefficients are determined by an additional constraint on the proportions of cases generated by the different sources of infection considered in the model. In particular, the contribution of each of the three sources of infection is set to 1/3. For **S** and **L** models, the transmission rates within households and schools/workplaces are kept fixed, while a specific rate is selected for the transmission in the communities, satisfying the above constraint on the proportions of cases generated by the different sources. Models including long-distance trips **M+T** and **L+T**) inherit transmission coefficients from the corresponding basic models.

This choice leads to within households and schools/workplaces transmission slightly smaller than in the respective basic models, because transmission during trips occurs only by random contacts in the population. In Table 6 the estimated transmission rates used for the simulations are reported (model details are given in Text S1). For our choices of the transmission rates, the final proportion of cases generated by the three sources differs no more than 0.018 from 1/3. For each model and choice of the first generation index, an average of at least 20,000 runs were considered, to guarantee a sufficiently accurate estimate of the relative transmission parameters.

**Table 6:** Transmission rates

*G_0_*	Model	β*_h_*	β*_s_*	β*_w_*	β*_u_*
**1.1**	**M**	0.4	1.4	0.7	0.2
	**M+T**	0.4	1.4	0.7	0.2
	**L**	0.4	1.4	0.7	0.32
	**L+T**	0.4	1.4	0.7	0.32
	**S**	0.4	1.4	0.7	0.22
**1.4**	**M**	0.5	2.0	1.0	0.25
	**M+T**	0.5	2.0	1.0	0.25
	**L**	0.5	2.0	1.0	0.37
	**L+T**	0.5	2.0	1.0	0.37
	**S**	0.5	2.0	1.0	0.29
**1.7**	**M**	0.6	2.6	1.3	0.3
	**M+T**	0.6	2.6	1.3	0.3
	**L**	0.6	2.6	1.3	0.48
	**L+T**	0.6	2.6	1.3	0.48
	**S**	0.6	2.6	1.3	0.36

Transmission rates estimated for the five models considered and for three different values of the first generation index. β*_h_*, β*_s._* and β*_w_* are the transmission rates in households, schools and workplaces respectively. β*_u_* is the transmission rate for unstructured contacts (details are given in Text S1).

## Supporting Information

Text S1 Supporting text
(0.11 MB PDF)

Figure S1 a Hierarchical structure of municipalities (dashed lines, M), provinces (solid thin lines, P) and regions (solid thick lines, R). b Models M and M+T: the social network of contacts of an individual living in municipality M123 and traveling to municipality M241 consists of all the individuals living in the two municipalities and the individuals traveling to one of the two municipalities (small filled circles).
(0.16 MB TIF)

Figure S2 Pseudocode of the algorithm employed for generating individuals, assigning age an co-locating individuals in households.
(0.26 MB TIF)

Figure S3 a Population data by municipality: colors ranging from dark brown (municipalities with less than 1,000 inhabitants) to light brown (more than 1,000,000 inhabitants, Rome and Milan) represent number of individuals on a *log^10^* scale. b Age distribution from census data (blue) and simulated (red). c As in b but showing household size. d Proportion of workers for class of workplace from industry census (blue) and simulated (red). e Commuting destinations of workers from census data (blue) and simulated (red). Symbols are defined in Sec. Sec. 1.4 in Text S1. f As in e but showing commuting destinations of students. Census data are available only for individuals of age > = 15 while simulated data refers to all students.
(0.64 MB TIF)

Figure S4 Blue curve: cumulative probability of commuting at a certain distance for the population simulated in the model (as obtained from census data). Red curve: cumulative probability of commuting at a certain distance as obtained by employing the kernel function in Eq. 3 in Text S1 with a = 3.6 and b = 1.9.
(0.12 MB TIF)

Table S1 Final attack rates
(0.02 MB PDF)

Table S2 Basic reproductive numbers
(0.02 MB PDF)

Table S3 Peak day
(0.02 MB PDF)

Table S4 Household types
(0.01 MB PDF)

Table S5 Household size by type
(0.02 MB PDF)

Movie S1 Spatiotemporal dynamics: infection is seeded in Cagliari (Sardinia island) and G^0^ = 1.7. Colored areas (model M in orange, M+T in red, L in cyan, L+T in blue, and S in green) indicate presence of at least one infected, infectious or removed individual.
(0.16 MB AVI)

Movie S2 Spatiotemporal dynamics: infection is seeded in Rome and G^0^ = 1.7. Colored areas (model M in orange, M+T in red, L in cyan, L+T in blue, and S in green) indicate presence of at least one infected, infectious or removed individual.
(0.16 MB AVI)
