# Supplementary material for: The Impact of the Unstructured Contacts Component in Influenza Pandemic Modeling
Source: PLoS One. 2008 Jan 30;3(1):e1519. doi: 10.1371/journal.pone.0001519 (PMC3278282; doi:10.1371/journal.pone.0001519)
Supplement: Text S1 — Supporting text (0.11 MB PDF) [file pone.0001519.s001.pdf]

The impact of the unstructured contacts component in influenza pandemic modeling

Supporting Information

Marco Ajelli, Stefano Merler

Contents

1 Structured contacts **1**
1.1 Population data . . . 1
1.2 Households . . . 1
1.3 Employment . . . 2
1.4 Commuting . . . 2

2 Disease transmission model **2**
2.1 Unstructured contacts . . . 3

3 Sensitivity analysis **4**

References **6**

1 Structured contacts

1.1 Population data

Population data of Italy (56,995,744 individuals) are obtained from census data [1] (382,534 census sections). Individuals are hierarchically grouped by municipalities (8,101), provinces (103) and regions (20), according to the political borders of the study area (see Fig. S1a). This choice is determined by the possibility of employing nationwide commuting data, described in Sec. 1.4, organized at the same level of detail. Number of individuals by municipality ranges from 33 to 2,546,804 (Rome municipality) with an average of 7,035 and a standard deviation of 39,326 (see Fig. S3a). Only 42 municipalities contain more than 100,000 individuals (6 of which contain more than 500,000), while 1,971 contain less than 1,000 individuals (37 of which less than 100).

1.2 Households

Census data on age structure and household type and size are jointly used with survey data [2] to randomly assign age and co–locate individuals in households. These data refer to the analysis of 19,227 households, corresponding to approximately 0.1% of the Italian households. Nine different types of household are considered: they are described in Table S4. Their size is

reported in Table S5. Note that the diction "without children" indicates both families without children or with children which do not live with the parents anymore.

The following additional constraints are also considered:

C1 any household must contain at least 1 adult (age ≥ 18);

C2 the age of any child is between 43 and 18 years less than that of the youngest parent;

C3 spouses age differs by no more than 15 years.

The algorithm employed for generating individuals, assigning age an co-locating individuals in households is described in Fig. S2. A comparison between real and simulated age structure is reported in Fig. S3b. A comparison between real and simulated data on household size is reported in Fig. S3c.

1.3 Employment

Demographic, school [3, 4] and industry [5] census data are used for randomly assigning an employment category to each individual on the basis of age. Population is structured as follows: 20,559,595 workers (862,552 of which employed as teachers, and thus employed in schools), 11,360,556 students and 25,084,274 unemployed or retired. Students are deterministically assigned to a specific school type (6 types, from nursery school to university) on the basis of age. Workers are assigned to a random workplace type (7 types, depending on the workplace size, i.e., the number of employees, see Fig. S3d).

1.4 Commuting

Commuting destination are assigned to fit available commuting data [1]. In particular, for each municipality the proportions are available of individuals older than 15 years old working or attending school in N) in the municipality of residence or traveling P) within the province they live in, R) outside the province but within the region, S) outside the region. We allow younger students to travel only within the province they live in. A comparison between simulated and real population commuting data is given in Fig. S3e-f.

2 Disease transmission model

The disease transmission is described by an individual-based model consisting of the following epidemic states: susceptible, exposed (i.e. the infected but not yet infectious individuals), infectious (they can be severe or mild cases) and recovered.

By adopting a similar notation as in [6, 7], for each individual i we define H_i as the set of the n_i individuals belonging to the same household of the individual i and L_i^j as the set of the m_i^j individuals attending the same school (index $j = 1, \ldots, 6$ identifies school type) or sharing the same workplace (index $j = 7, \ldots, 13$ identifies workplace type) of the individual i.

Any susceptible individual i has a probability of becoming infected equal to

$$p_i \quad = \quad 1 - e^{-\lambda_i \Delta t}, \tag{1}$$

where $\Delta t = 0.5$ days is the time step of the simulation and λ_i is the instantaneous risk of infection. The latter is the sum of the risks coming from the three source of infections:

1. contacts with infectious members of the household (first term in Eq. 2),
2. contacts with infectious individuals working in the same workplace or attending the same school (second term in Eq. 2),
3. unstructured contacts with infectious individuals in the population (third term in Eq. 2, defined in Sec. 2.1).

$$\begin{aligned}
\lambda_i &= \sum_{k|H_k=H_i} \frac{I_k \beta_h \left[1 + C_k(\omega - 1)\right]}{n_i^{\alpha}} \\
&+ \sum_{j,k|L_k^j=L_i^j} \frac{I_k \beta_p^j \left[1 + C_k(\omega \psi_p^j(t-\tau_k) - 1)\right]}{m_i^j} \\
&+ U_i
\end{aligned} \tag{2}$$

The terms in Eq. (2) are defined as follows:

- $I_i = 1$ if individual i is infectious, 0 otherwise;
- β_h is the within–household transmission coefficient, β_p^j are the within–school/workplace transmission coefficients, and β_u is the unstructured transmission parameter (explicitly employed for modeling transmission by unstructured contacts U_i, see Sec. 2.1).
- $C_k = 1$ for severe cases (we suppose the 50% of cases to be severe), 0 otherwise. Since $\omega = 2$, the infectiousness of severe cases doubles the one of mild cases (as in [6]);
- $\alpha = 0.8$ scales the household transmission rates with household size (as in [6]);
- τ_i is the time individual i became infectious;
- $\psi_p^j(T)$ is a function accounting for induced absenteeism and it is defined as follows: if $T > 0.5$ (the minimum time for recognizing the infection) $\psi_p^j(T)$ is set to 0.1 for $j = 1, 2$, 0.2 for $j = 3, 4$, 0.25 for $j = 5$ and 0.5 for $j = 6, \ldots, 13$, 1 otherwise;

According to the literature, latent period is set to 1.5 ± 0.5 days (as in [6, 8]) and invectiveness period is set to 4 days (as in [9, 8]).

2.1 Unstructured contacts

The third term of Eq. (2) accounts for the risk of infection coming from the unstructured contacts. We consider different models of transmission by unstructured contacts.

S : unstructured contacts by a spatially explicit model:

$$U_i = \sum_{k=1}^{N} \frac{I_k \beta_u K(d_{ik}) \left[1 + C_k(\omega - 1)\right]}{\sum_{j=1}^{n} K(d_{ij})} ,$$

where N is the size of the population, d_{ik} is the geographic (euclidean) distance between individuals i and k, and

$$K(d_{ik}) = \frac{1}{1 + (\frac{d_{ik}}{a})^b} . \quad (3)$$

The kernel parameters employed in the present work are $a = 3.6$ and $b = 1.9$. They can be estimated by fitting the cumulative probability of travel for a certain distance as obtained from commuting data (see Fig. S4).

L : unstructured contacts within the local community:

$$U_i = \sum_{k \in S_i} \frac{I_k \beta_u \left[1 + C_k(\omega - 1)\right]}{s_i} ,$$

where S_i is the set (of cardinality s_i) of individuals living in the same municipality where individual i lives in.

M : unstructured contacts within the "commuting community":

$$U_i = \sum_{k \in S_i^l} \frac{I_k \beta_u \left[1 + C_k(\omega - 1)\right]}{s_i^l} + \sum_{k \in S_i^c} \frac{I_k \beta_u \left[1 + C_k(\omega - 1)\right]}{s_i^c} ,$$

where S_i^l is the set (of cardinality s_i^l) of individuals living or commuting to the same municipality where individual i lives in and S_i^c is the set (of cardinality s_i^c, set to 0 for non traveling individuals) of individuals living or commuting to the same municipality where individual i commutes to (see Fig. S1b).

Moreover, we consider two additional models that include occasional long–distance trips (as in [9]) in models **L** and **M**, called **L+T** and **M+T** respectively. In particular, the daily probability of spending a day in a (randomly chosen) community other than that of residence and school/work, is 10/365. In these periods, transmission within household, school and workplace is not allowed; thus, the only non-zero term in Eq. (2) is U_i.

The model **L** can be written in the same form of model **S** by defining a "behavioral distance" $d_{ik} = 1$ if individual k lives in the same municipality where i lives in, ∞ otherwise. A similar argument holds for the model **M**.

In **M** and **M+T**, unstructured contacts are modeled on the basis of commuting data, to capture both the short distance (i.e., within the community of residence), and the medium to long distance population mobility. Indeed, differently from [6], where all the individuals are potential contacts of an infectious individual and the risk of being infected decreases with the distance, the risk of infection depends on the behavior of the individuals rather than on relative distance among them.

3 Sensitivity analysis

In the Main Text we observed that the introduction of occasional long–distance trips substantially decreases the final attack rate of both the **M+T** and the **L+T** models since, in our

implementation, transmission is not allowed within household and within school/workplace during long–distance trips. Here we show the effect of varying the number of days spent for occasional long–distance trips, keeping fixed the seeding municipality (Turin).

The attack rate decreases drastically by increasing the number of travel days (see Tab S1), especially for low values of G_0. For $G_0 = 1.1$, a reduction of 50.4% and 42.7% has been observed by increasing the number of travel days from 5 to 30 in models **M+T** and **L+T** respectively. These percentages become 15.6% and 14.4% for $G_0 = 1.4$ and 8.8% and 7% for $G_0 = 1.7$. However, it is worth noting that this is due to a reduction in the actual value of G_0 which reflects in a reduction of R_0 (see Tab S2). In fact, for any given number of days spent for long–distance trips it is always possible to choose a value of transmission parameter β_u such that the attack rate does not change with respect to the models without long–distance trips (**M** and **L**). However, in this case the contribution of each of the three sources of infection (set to 1/3 in the models **M** and **L**) consequently will change. In general, for any given number of travel days, the attack rates of models **L+T** are larger than those of models **M+T**.

By varying the number of travel days in models **M+T** and **L+T**, significant differences are observed in the peak day only for low values of G_0 (see Tab. S3). This behaviour is related to the observed percentages of attack rate reduction. In particular, the larger the number of travel days, the later the peak day.

As regards the effect of varying the kernel parameter b in models **S**, no significant differences were observed in terms of the macroscopic epidemiological features considered in this work (see Tab. S1, S2 and S3). However, as shown in [6, 7], the choice of kernel parameter b has a great impact in evaluating geographically targeted containment/mitigation strategies.

References

[1] Italian Institute of Statistics (ISTAT). XIV Censimento generale della popolazione e delle abitazioni. `http://dawinci.istat.it/MD/`, 2001.

[2] Italian Institute of Statistics (ISTAT). Strutture familiari e opinioni su famiglia e figli.`http://www.istat.it/dati/catalogo/20060621_03/`, 2003.

[3] Italian Ministry of University and Research (MIUR). La scuola in cifre. `http://www.miur.it/ustat/documenti/pub2005/`, 2005.

[4] Italian Ministry of University and Research (MIUR). L'università in cifre. `http://www.miur.it/ustat/documenti/pub2005/`, 2005.

[5] Italian Institute of Statistics (ISTAT). VIII Censimento generale dell'industria e dei servizi. `http://dwcis.istat.it/cis/index.htm`, 2001.

[6] N. M. Ferguson, D. A. Cummings, S. Cauchemez, C. Fraser, S. Riley, A. Meeyai, S. Iamsirithaworn, and D. S. Burke. Strategies for containing an emerging influenza pandemic in Southeast Asia. *Nature*, 437:209–214, 2005.

[7] N. M. Ferguson, D. A. Cummings, C. Fraser, J. C. Cajka, P. C. Cooley, and D. S. Burke. Strategies for mitigating an influenza pandemic. *Nature*, 442:448–452, 2006.

[8] I. M. Jr Longini, A Nizam, S Xu, K Ungchusak, W Hanshaoworakul, D. A. Cummings, and M. E. Halloran. Containing pandemic influenza at the source. *Science*, 309(5737):1083–7, 2005.

[9] T. C. Germann, K. Kadau, I. M. Jr Longini, and C. A. Macken. Mitigation strategies for pandemic influenza in the United States. *Proc Natl Acad Sci USA*, 103(15):5935–4, 2006.
