# Supplementary figures and images for: The Impact of the Unstructured Contacts Component in Influenza Pandemic Modeling
Source: PLoS One. 2008 Jan 30;3(1):e1519. doi: 10.1371/journal.pone.0001519 (PMC3278282; doi:10.1371/journal.pone.0001519)

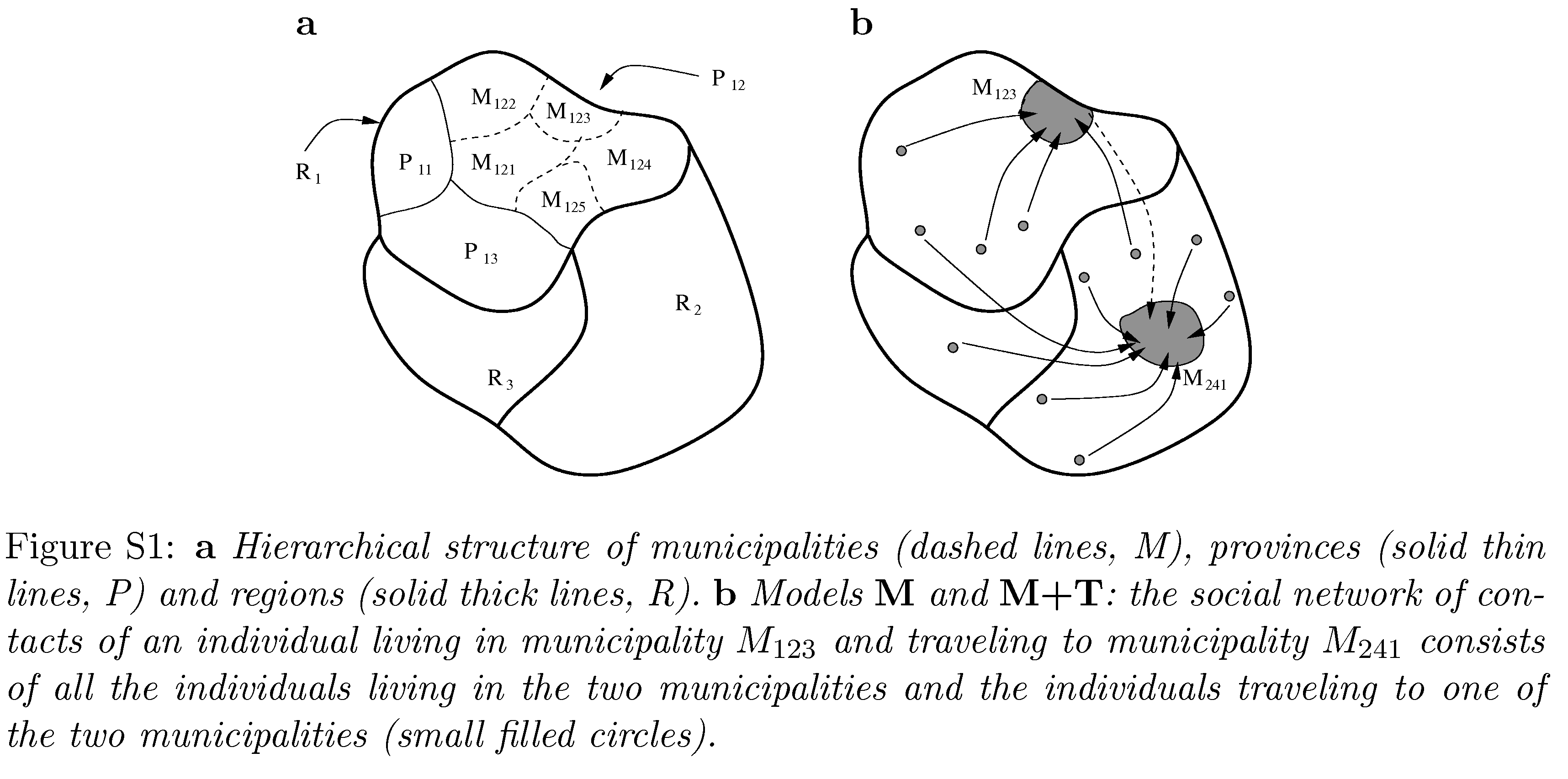

Supplement: Figure S1 — a Hierarchical structure of municipalities (dashed lines, M), provinces (solid thin lines, P) and regions (solid thick lines, R). b Models M and M+T: the social network of contacts of an individual living in municipality M123 and traveling to municipality M241 consists of all the individuals living in the two municipalities and the individuals traveling to one of the two municipalities (small filled circles). (0.16 MB TIF) [file pone.0001519.s002.tif]

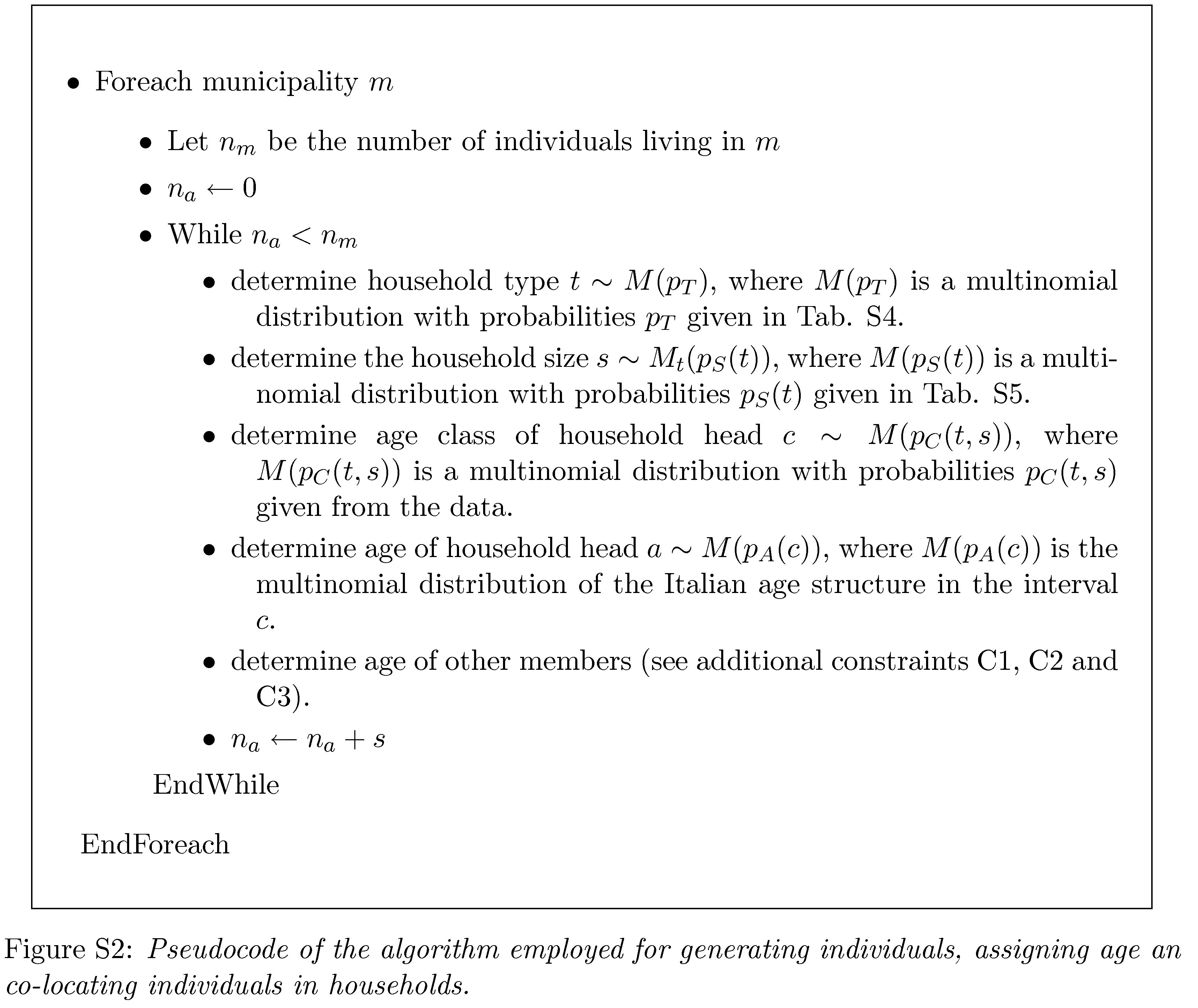

Supplement: Figure S2 — Pseudocode of the algorithm employed for generating individuals, assigning age an co-locating individuals in households. (0.26 MB TIF) [file pone.0001519.s003.tif]

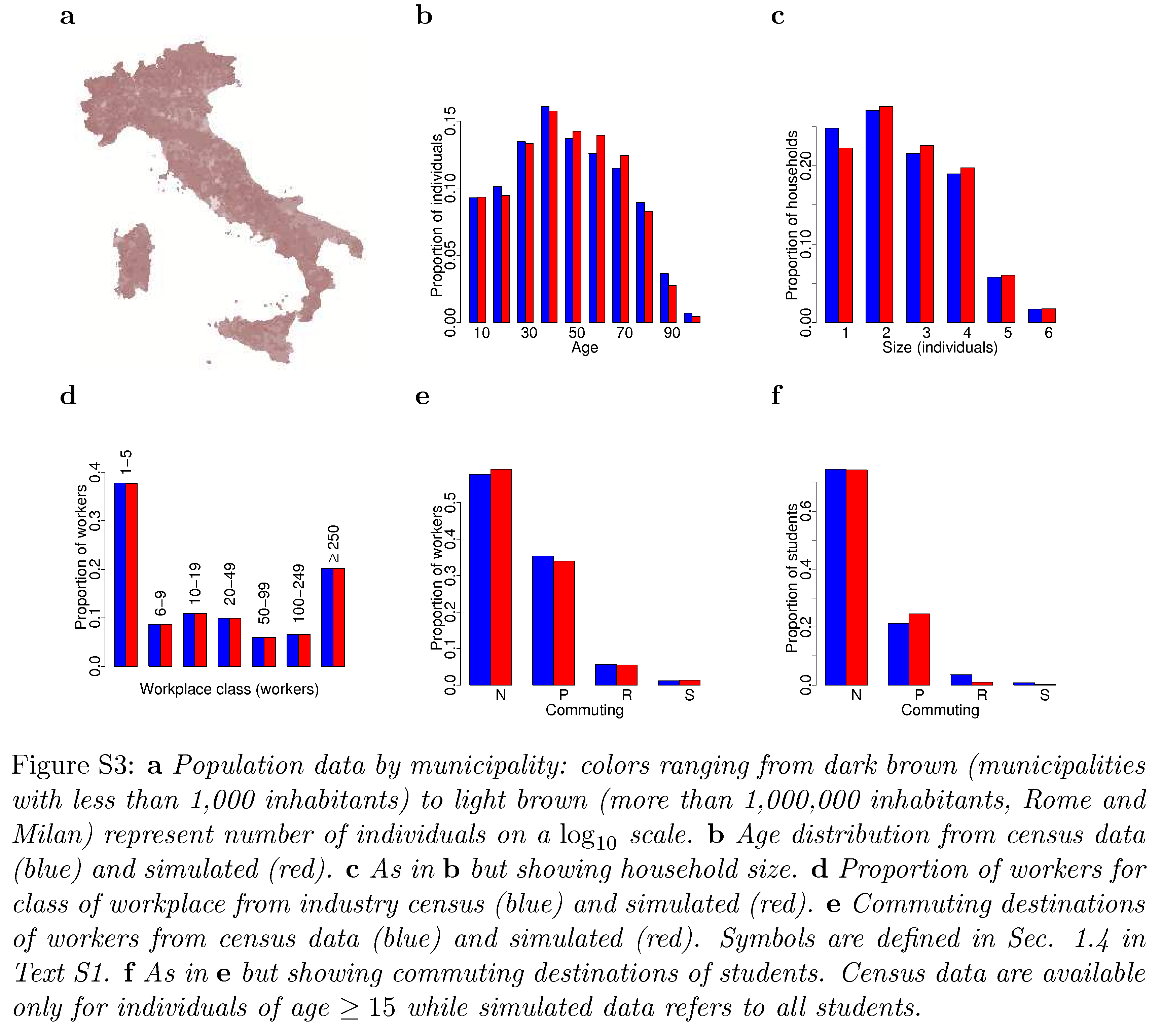

Supplement: Figure S3 — a Population data by municipality: colors ranging from dark brown (municipalities with less than 1,000 inhabitants) to light brown (more than 1,000,000 inhabitants, Rome and Milan) represent number of individuals on a log10 scale. b Age distribution from census data (blue) and simulated (red). c As in b but showing household size. d Proportion of workers for class of workplace from industry census (blue) and simulated (red). e Commuting destinations of workers from census data (blue) and simulated (red). Symbols are defined in Sec. Sec. 1.4 in Text S1. f As in e but showing commuting destinations of students. Census data are available only for individuals of age > = 15 while simulated data refers to all students. (0.64 MB TIF) [file pone.0001519.s004.tif]

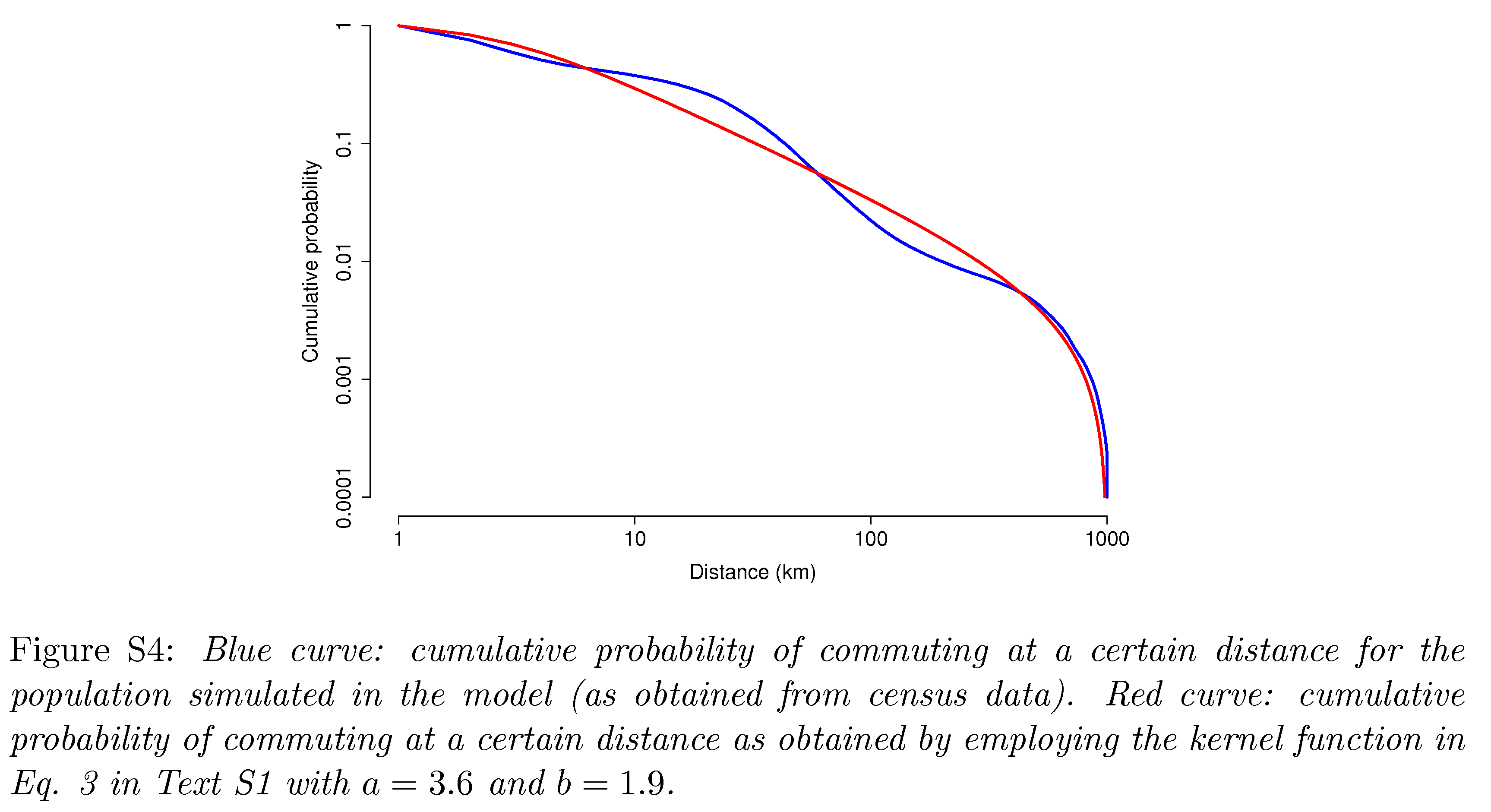

Supplement: Figure S4 — Blue curve: cumulative probability of commuting at a certain distance for the population simulated in the model (as obtained from census data). Red curve: cumulative probability of commuting at a certain distance as obtained by employing the kernel function in Eq. 3 in Text S1 with a = 3.6 and b = 1.9. (0.12 MB TIF) [file pone.0001519.s005.tif]
