# Supplementary material for: The Impact of the Unstructured Contacts Component in Influenza Pandemic Modeling
Source: PLoS One. 2008 Jan 30;3(1):e1519. doi: 10.1371/journal.pone.0001519 (PMC3278282; doi:10.1371/journal.pone.0001519)
Supplement: Table S1 — Final attack rates (0.02 MB PDF) [file pone.0001519.s006.pdf]

Table S1: *Final attack rate (with standard deviation) for different G_0 values obtained by varying the number of days spent for occasional long–distance trips in the models* **M+T** *and* **L+T** *and by varying the kernel parameter b (see Eq.3) in models* **S**.

Model	days	b	AR		
			$G_0 = 1.1$	$G_0 = 1.4$	$G_0 = 1.7$
**M+T**	5	–	23.6 (0.029)	49.2 (0.021)	63.5 (0.010)
	10	–	21.5 (0.037)	47.7 (0.015)	62.4 (0.014)
	20	–	16.9 (0.103)	44.6 (0.015)	60.2 (0.014)
	30	–	11.7 (0.767)	41.5 (0.016)	57.9 (0.012)
**L+T**	5	–	24.8 (0.040)	49.3 (0.016)	66.7 (0.010)
	10	–	22.6 (0.026)	47.9 (0.019)	65.7 (0.012)
	20	–	18.8 (0.053)	44.9 (0.019)	63.8 (0.011)
	30	–	14.2 (1.086)	42.2 (0.014)	62.0 (0.015)
**S**	–	0.6	19.6 (0.030)	48.7 (0.015)	64.8 (0.010)
	–	1.2	19.6 (0.046)	48.7 (0.019)	64.8 (0.010)
	–	1.9	19.6 (0.065)	48.7 (0.038)	64.8 (0.015)
	–	2.6	19.6 (0.040)	48.7 (0.016)	64.8 (0.010)
	–	5.2	19.6 (0.061)	48.7 (0.018)	64.8 (0.013)
