# Supplementary material for: The Impact of the Unstructured Contacts Component in Influenza Pandemic Modeling
Source: PLoS One. 2008 Jan 30;3(1):e1519. doi: 10.1371/journal.pone.0001519 (PMC3278282; doi:10.1371/journal.pone.0001519)
Supplement: Table S2 — Basic reproductive numbers (0.02 MB PDF) [file pone.0001519.s007.pdf]

Table S2: *Basic reproductive numbers R_0 (with standard deviation) for different G_0 values obtained by varying the number of days spent for occasional long–distance trips in the models* **M+T** *and* **L+T** *and by varying the kernel parameter b (see Eq.3) in models* **S**.

Model	days	b	R_0		
			$G_0 = 1.1$	$G_0 = 1.4$	$G_0 = 1.7$
**M+T**	5	–	1.34 (0.020)	1.75 (0.010)	2.11 (0.010)
	10	–	1.29 (0.022)	1.71 (0.010)	2.11 (0.013)
	20	–	1.19 (0.037)	1.67 (0.011)	2.04 (0.010)
	30	–	1.19 (0.040)	1.63 (0.018)	1.97 (0.011)
**L+T**	5	–	1.27 (0.029)	1.68 (0.015)	2.08 (0.010)
	10	–	1.29 (0.025)	1.67 (0.011)	2.10 (0.011)
	20	–	1.27 (0.032)	1.61 (0.018)	2.02 (0.011)
	30	–	1.21 (0.035)	1.56 (0.021)	1.97 (0.012)
**S**	–	0.6	1.25 (0.035)	1.73 (0.011)	2.13 (0.010)
	–	1.2	1.26 (0.027)	1.74 (0.011)	2.13 (0.011)
	–	1.9	1.27 (0.033)	1.72 (0.012)	2.14 (0.011)
	–	2.6	1.29 (0.038)	1.72 (0.010)	2.15 (0.012)
	–	5.2	1.28 (0.034)	1.73 (0.009)	2.17 (0.009)
