# Supplementary material for: The Impact of the Unstructured Contacts Component in Influenza Pandemic Modeling
Source: PLoS One. 2008 Jan 30;3(1):e1519. doi: 10.1371/journal.pone.0001519 (PMC3278282; doi:10.1371/journal.pone.0001519)
Supplement: Table S3 — Peak day (0.02 MB PDF) [file pone.0001519.s008.pdf]

Table S3: *Peak day (with standard deviation) for different G_0 values obtained by varying the number of days spent for occasional long–distance trips in the models* **M+T** *and* **L+T** *and by varying the kernel parameter b (see Eq.3) in models* **S**.

Model	days	b	Peak Day		
			$G_0 = 1.1$	$G_0 = 1.4$	$G_0 = 1.7$
**M+T**	5	–	273.3 (24.0)	148.2 (11.7)	101.6 (8.3)
	10	–	288.9 (25.4)	136.6 (10.2)	98.0 (6.8)
	20	–	344.3 (28.7)	149.1 (15.0)	99.0 (5.2)
	30	–	417.0 (12.7)	156.4 (13.7)	103.3 (7.6)
**L+T**	5	–	287.1 (18.2)	148.2 (11.7)	99.4 (6.4)
	10	–	388.3 (148.2)	142.8 (11.5)	98.9 (7.7)
	20	–	326.0 (26.7)	141.1 (10.2)	101.1 (6.9)
	30	–	416.3 (24.0)	147.9 (11.8)	104.9 (7.8)
**S**	–	0.6	315.6 (34.2)	126.3 (7.2)	88.5 (6.5)
	–	1.2	295.7 (21.3)	123.2 (8.8)	86.6 (6.3)
	–	1.9	298.3 (25.5)	126.4 (9.6)	89.6 (5.8)
	–	2.6	292.8 (26.1)	122.1 (14.0)	85.5 (6.6)
	–	5.2	286.3 (24.9)	129.2 (16.4)	87.4 (8.2)
