# Supplementary material for: The Impact of the Unstructured Contacts Component in Influenza Pandemic Modeling
Source: PLoS One. 2008 Jan 30;3(1):e1519. doi: 10.1371/journal.pone.0001519 (PMC3278282; doi:10.1371/journal.pone.0001519)
Supplement: Table S4 — Household types (0.01 MB PDF) [file pone.0001519.s009.pdf]

Table S4: *Percentage of different household types.* * *with additional household member.*

household type	percentage
single without children	25.8
single with children	7.9
single with children*	0.6
couple without children	19.7
couple without children*	1.3
couple with children	40.0
couple with children*	1.9
adults living together	1.6
more household groups	1.2
