# Supplementary material for: The Impact of the Unstructured Contacts Component in Influenza Pandemic Modeling
Source: PLoS One. 2008 Jan 30;3(1):e1519. doi: 10.1371/journal.pone.0001519 (PMC3278282; doi:10.1371/journal.pone.0001519)
Supplement: Table S5 — Household size by type (0.02 MB PDF) [file pone.0001519.s010.pdf]

Table S5: *Household size by type (in percentage).* * *with additional household member.*

household type	household size					
	1	2	3	4	5	≥ 6
single without children	100	0	0	0	0	0
single with children	0	68.9	25	6.1	0	0
single with children*	0	0	68.9	25	6.1	0
couple without children	0	100	0	0	0	0
couple without children*	0	0	100	0	0	0
couple with children	0	0	45.1	43.8	9.5	1.6
couple with children*	0	0	0	45.1	43.8	11.1
adults living together	0	78.5	21.5	0	0	0
more household groups	0	0	0	49.3	44.3	6.4
